# Influence of reinforcement on the fracture surface characteristics of multistage solutionized and precipitation-hardened ternary Al-Mg-Si hybrid composites

**DOI:** 10.1038/s41598-025-34581-4

**Published:** 2026-01-03

**Authors:** Manjunath Shettar, Nitesh Kumar, Girish Hariharan, Gowri shankar, Ananda Hegde

**Affiliations:** Department of Mechanical and Industrial Engineering , Manipal Institute of Technology, Manipal Academy of Higher Education , Manipal, 576104 India

**Keywords:** Al-Mg-Si alloy, Silicon carbide, Boron carbide, Artificial age hardening treatment, Hardness, Ultimate tensile strength, Fracture surface analysis, Materials engineering, Engineering, Materials science

## Abstract

This study investigates the effect of SiC, B₄C, and hybrid (SiC + B₄C) reinforcements on the fracture surface characteristics of multistage solutionized and precipitation-hardened Al-Mg-Si (Al 6061) alloy. Composites are fabricated using two-stage stir casting with SiC (1–3 wt%), B₄C (1–3 wt%), and hybrid reinforcements (1–2 wt% each). As-cast (AC) samples are subjected to single-stage solutionizing (SSS) and multistage solutionizing (MSS), followed by artificial aging at 100 and 200 °C. Results show that MSS-aged samples exhibit a peak hardness of 222 VHN, a 177% improvement over AC (80 VHN). Similarly, UTS increases from 128 MPa (AC) to 287 MPa in hybrid composites, marking a 124% enhancement. Fractography shows that unreinforced Al 6061 failed mainly in a ductile mode with coarse dimples, while monolithic composites exhibit mixed ductile–brittle features. Hybrid composites exhibit refined dimples and strong interfacial bonding, thereby enhancing crack resistance. AAHT-treated samples show a shift toward brittle-dominant failure. The combined effect of dual reinforcements and MSS treatment enhances both strength and fracture resistance, demonstrating the potential of Al 6061 hybrid composites for lightweight structural applications.

## Introduction

Aluminum (Al) and its composites have emerged as essential materials in various industrial sectors, particularly in automotive, aerospace, and marine applications, due to their remarkable combination of lightweight characteristics and mechanical properties^1^. Compared to steels, aluminum exhibits a superior strength-to-weight ratio, which enables significant weight reduction without compromising structural integrity^2^. This advantage has driven extensive research and development of aluminum-based composites tailored to meet the stringent mechanical and thermal requirements of modern engineering components^3^. In applications such as pistons, cylinder heads, gearboxes, brackets, housings, mounting structures, and crankcases, aluminum composites are required to exhibit an optimal balance of mechanical properties, particularly hardness and UTS.

The mechanical performance of aluminum composites is primarily governed by the type, size, distribution, and volume fraction of the reinforcement particles incorporated within the matrix^4^. Among various reinforcement materials, hard ceramic particulates are widely preferred because of their ability to enhance strength, stiffness, and wear resistance^5^. Reinforcements such as silicon carbide (SiC), boron carbide (B₄C), and aluminum oxide (Al₂O₃) have been extensively studied for their role in improving the hardness and tensile behavior of aluminum matrices^6^.

Alaneme et al.^7^ investigate the fractography of tensile specimens of as-cast and age-hardened aluminum composites reinforced with SiC particulates (30 μm, 3–12 wt%). The samples undergo solution treatment at 560 °C for 2 h, followed by water quenching. Artificial aging is then performed at 180 °C for 3 h. The composites exhibit a 30–40% increase in tensile strength (up to 150 MPa) compared to the monolithic alloy. This enhancement is attributed to a decrease in interparticle spacing, which impedes dislocation mobility and promotes the formation of coherent Mg₂Si precipitates.

Kenneth et al.^8^ examine the production quality and age-hardening behavior of Al6063/SiC particulate composites (30 μm) fabricated via stir casting using borax as an additive. The specimens undergo solution treatment at 560 °C for 2 h, followed by water quenching. Aging is then carried out at 180, 190, and 200 °C for 30 to 360 min. Results show that the monolithic alloy achieves a peak hardness between 60 and 85 VHN, while the composites attain significantly higher values, ranging from 100 to 120 VHN.

Bommana et al.^9^ evaluate the mechanical behavior of AA6061 hybrid composites reinforced with a fixed 6 wt% of SiC and B₄C in varying ratios. Increasing B₄C content leads to a notable enhancement in hardness, while a balanced 3:3 wt% ratio of SiC and B₄C yields the highest tensile and yield strengths. Composites with higher B₄C content exhibit improved elongation, attributed to Mg-induced refinement of the Mg₂Si eutectic phase. The study highlights the role of optimized reinforcement distribution in enhancing the strength, hardness, and ductility of materials.

Baradeswaran et al.^10^ explore the effect of B₄C reinforcement on the tribological properties of aluminum composites synthesized using potassium fluotitanate (K₂TiF₆) to improve wettability. The study reveals improvements in hardness, tensile strength, and wear resistance with increased B₄C content. The increased hardness is linked to enhanced strain energy at the particle–matrix interface, while improved tensile strength is due to effective load transfer mechanisms. The observed wear resistance stems from the formation of a mechanical mixed layer during wear.

Auradi et al.^11^ develop B₄C-reinforced Al6061 composites using a two-step stir casting method. Preheating the mixture of K₂TiF₆ and B₄C during stirring ensures better wettability and uniform particle dispersion compared to single-step casting. The addition of B₄C improves mechanical properties, particularly hardness and tensile strength, though it reduces ductility. The enhanced hardness is attributed to the resistance offered by hard B₄C particles to plastic deformation, as well as the associated increase in strain energy.

Srinivas et al.^12^ demonstrate that precipitation hardening significantly improves the mechanical properties of Al alloys, particularly when combined with ceramic reinforcements. Through solutionizing and subsequent aging, solute-rich precipitates such as θ′-Al₂Cu and θ′′-Al₃Cu form, thereby increasing hardness. Multistage solution heat treatment (MSS) achieves better homogenization and finer precipitate distribution than single-stage treatment, leading to a reported 150% increase in hardness. Reinforcement presence further contributes through dislocation strengthening and grain refinement.

Mohamed et al.^13^ find that multistage solutionizing treatment (495 °C for 2 h + 515 °C for 4 h) yields better strength and ductility than a single-stage treatment at 495 °C for 8 h. However, they caution that the second-stage temperature should remain below 520 °C to avoid property degradation. Their aging process involves solutionizing at 490–540 °C for 8 h, quenching at 60 °C, and aging at 155 °C for 5 h. They observe that temperatures above 530 °C in two-stage treatments negatively impact UTS and yield strength (YS).

Recent advances in hybrid and particle-reinforced light-metal systems have demonstrated that combining ceramic or carbon-based reinforcements with multistage heat treatment and severe plastic deformation can remarkably enhance strength and ductility. Huang et al.^14^ report that graphene-reinforced AZ91 magnesium composites subjected to T6 heat treatment and Equal Channel Angular Pressing (ECAP) exhibit refined microstructure and superior mechanical properties due to synergistic reinforcement–matrix interaction and dynamic recrystallization.

Sadananda et al.^15^ report significant improvement in the mechanical properties of AA6061 hybrid composites through artificial aging at 100 °C. The presence of reinforcements and optimized aging conditions leads to increased hardness and tensile strength. Fracture surface analysis reveals a mixed-mode failure, characterized by features such as elongated dimples and tear ridges. These enhancements result from increased dislocation density, strain hardening, and adequate precipitation.

Han et al.^16^ analyze the UTS of Al-Si-Cu-Mg alloys subjected to single-stage (450–490 °C for 4–8 h), multistage (500–520 °C), and triple-stage (> 520 °C) solution heat treatments. They find that triple-stage treatments lower tensile properties due to incipient melting and coarsening of silicon particles. Consequently, they recommend avoiding triple-stage heat treatments for industrial-grade Al alloys containing 0.3 wt% Mg.

Sokolowski et al.^17^ compare single and multistage solution treatments for 319 Al-alloy, including strontium-modified Cu-rich eutectic behavior. They find that a single-stage treatment at 495 °C for 8 h is insufficient for dissolving Cu-rich phases. In contrast, a multistage regime (495 °C for 2 h + 515 °C for 4 h), followed by quenching at 74 °C and aging at 250 °C for 3 h, results in superior mechanical properties.

Zhu et al.^18^ emphasize the importance of solution heat treatment (SHT) for homogenizing α-Al dendrites and altering precipitation behavior. Treatment at 540 °C for 10 min effectively homogenizes the α-Al phase and modifies silicon morphology. T6-treated alloys show significantly improved mechanical properties due to Mg₂Si formation and eutectic Si spheroidization.

Despite substantial research on Al6061-based composites, a critical knowledge gap remains in understanding mechanical property enhancement under advanced heat treatment regimes, particularly multistage solutionizing (MSS) followed by AAHT. While several studies address the individual effects of SiC and B₄C reinforcements, their combined influence under controlled MSS and AAHT conditions remains underexplored. Additionally, fracture surface analysis post-AAHT is scarcely reported for both monolithic and hybrid composites.

This study presents a novel and comprehensive investigation into the synergistic effects of silicon carbide (SiC), boron carbide (B₄C), and their hybrid (SiC + B₄C) reinforcements on the microstructural evolution, mechanical performance, and fracture characteristics of Al6061 matrix composites. The composites are subjected to optimized single-stage solutionizing (SSS) and multistage solutionizing (MSS) heat treatments, followed by AAHT, to elucidate the correlation between thermal treatment parameters, reinforcement morphology, and the resultant mechanical response. The hybrid composite under MSS conditions exhibits superior strength and hardness, attributed to homogeneous precipitate distribution, refined grain structure, and enhanced interfacial bonding. A comparative analysis of as-cast, SSS, and MSS-treated specimens highlights the novelty of integrating hybrid ceramic reinforcements with a tailored multistage solutionizing route to achieve an optimal balance of strength and ductility. The outcomes of this work provide critical insights for developing thermally engineered, high-performance Al6061-based composites for advanced structural and aerospace applications.

## Materials and methodology

In the current work, Al6061 alloy was used as the matrix; the chemical composition of Al6061 is presented in Table 1 below. It was procured from Laxmi Metal, Coimbatore, India. B_4_C and SiC with an average particle size of 36 μm were used as reinforcements.

**Table 1:** Standard and actual chemical composition of al 6061 alloy.

Alloying element	Standard (wt%)	Actual (wt%)
Silicon (Si)	0.40–0.80	0.54
Copper (Cu)	0.15–0.40	0.26
Magnesium (Mg)	0.80–1.20	0.99
Iron (Fe)	Max 0.70	0.64
Titanium (Ti)	Max 0.15	0.14
Chromium (Cr)	0.04–0.35	0.27
Aluminum	Balance	

**Fig. 1:**
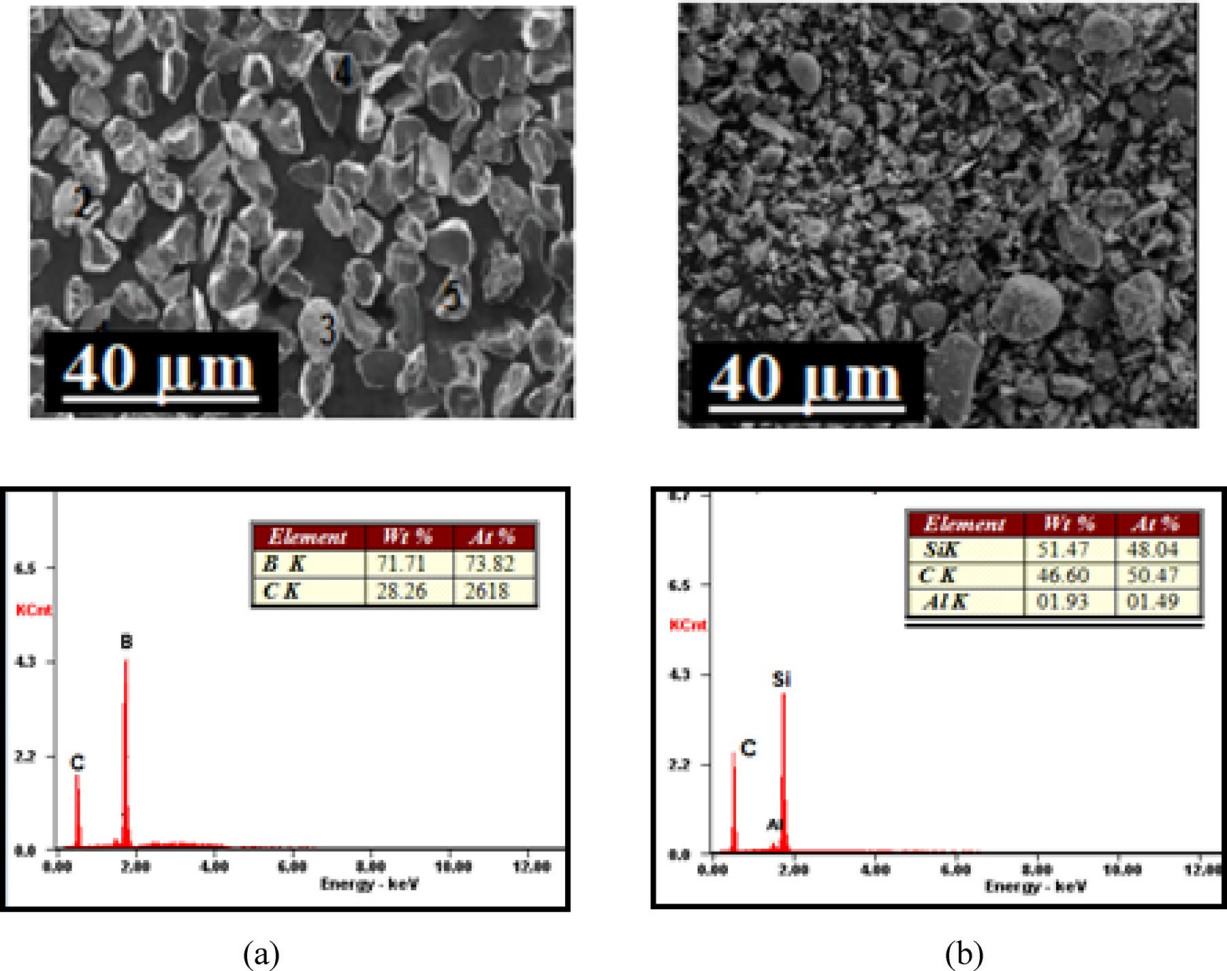
SEM and EDAX reports of (**a**) B_4_C and (**b**) SiC reinforcement powders.

**Fig. 2:**
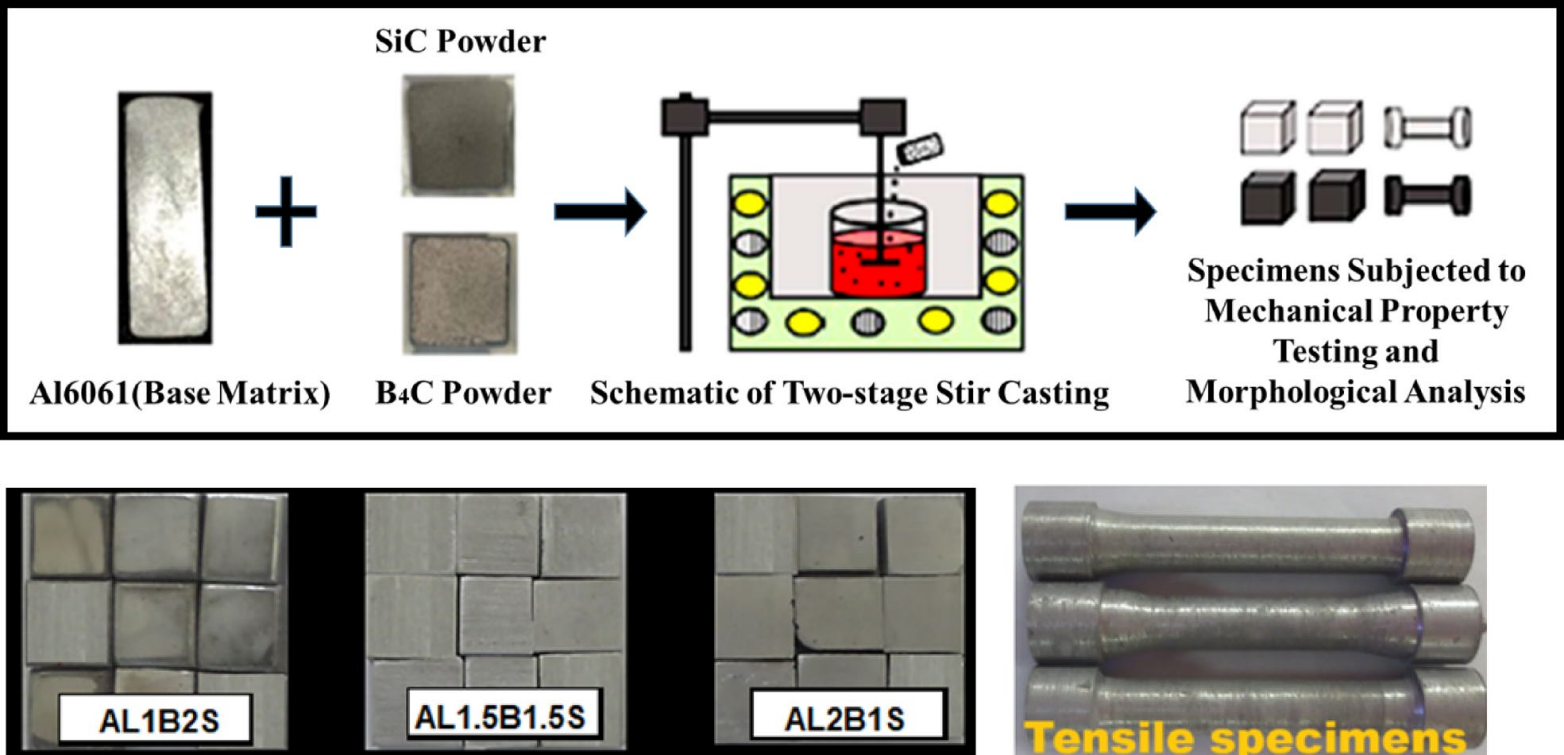
Schematic representation of the stir casting setup employed in the present study, along with the fabricated test specimens.

EDAX and SEM of the reinforcement powders used for the present work are displayed in Fig. 1. Monolithic and hybrid composites were fabricated using a two-stage stir-casting method^19^. Before the addition of reinforcements, they were preheated at 300 °C for 30 min to improve the wettability and uniform dispersion of the reinforcements^20^. Once the casting was performed, the molten mix was emptied into molds (heated to 500 °C for 2 h) for improved solidification. Figure 2 illustrates a schematic representation of the stir casting setup used in the present study, along with the fabricated test specimens. The short forms assigned to the cast composites are displayed in Table 2.

Composite samples were divided into four batches. The first batch was subjected to SSS, where specimens [S1] were soaked at 495 °C for 8 h, followed by hot water quenching at 60 °C and then AAHT at 100 °C. The second batch [S2] was subjected to SSS and AAHT at 200 °C. The third batch [M1] was subjected to MSS, where samples were soaked at 495 and 520 °C for 2 and 4 h, followed by hot water quenching at 60 °C and AAHT at 100 °C. The fourth batch [M2] was subjected to MSS and AAHT at 200 °C. Artificial aging was performed at these temperatures (100 and 200 °C) for various durations^12^. A hardness comparison was made between AC and precipitation-hardened (peak-aged) samples of Al 6061 and its composites. As per ASTM E384, a hardness test was performed using a Micro Vickers Hardness Tester (MODEL MMT X 7 A). The tensile specimens were prepared in accordance with ASTM E8 sub-size flat specimen standards. The gauge section dimensions were as follows: Gauge length: 24 mm, diameter: 6 mm, Overall length: 48 mm. Number of Specimens Tested: Three tensile specimens were tested for each material condition, and the average UTS was reported. Test Speed: Tensile tests were conducted on an Electronic Universal Testing Machine (Model: PC-2000/605/06) with a crosshead speed of 1 mm/min, in accordance with the recommendations of ASTM E8 for sub-size specimens. FSA was performed on the samples using SEM images [ZEISS EV018].

**Table 2:** Short forms of cast composites (wt% of reinforcements).

S. No.	Composite (wt% of reinforcements)	Short form
1	Al 6061 and B_4_C (1 wt%)	AL1B
2	Al 6061 and B_4_C (2 wt%)	AL2B
3	Al 6061 and B_4_C (3 wt%)	AL3B
4	Al 6061 and SiC (1 wt%)	AL1S
5	Al 6061 and SiC (2 wt%)	AL2S
6	Al 6061 and SiC (3 wt%)	AL3S
7	Al 6061, B_4_C (1 wt%), and SiC (2 wt%)	AL1B2S
8	Al 6061, B_4_C (1.5 wt%), and SiC (1.5 wt%)	AL1.5B1.5 S
9	Al 6061, B_4_C (2 wt%), and SiC (1 wt%)	AL2B1S

## Results and discussion

### Microstructure evaluation of as-cast specimens

**Fig. 3:**
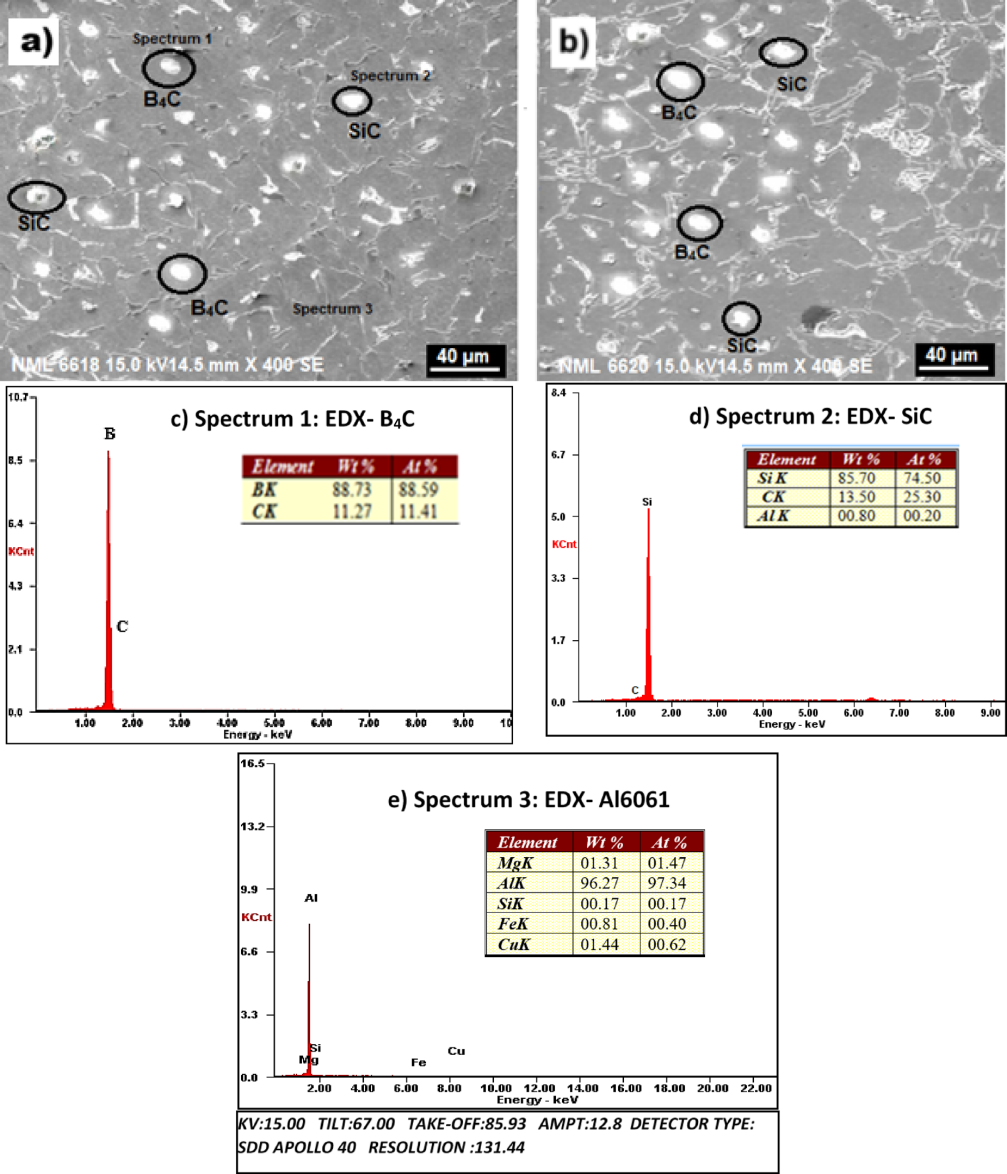
SEM micrographs (**a**, **b**) of the as-cast Al6061–2B1S hybrid composite and corresponding EDX spectra of (**c**) B₄C, (**d**) SiC, and (**e**) Al6061 alloy matrix.

Uniform dispersion of reinforcement particles within the metal matrix is crucial for achieving enhanced mechanical properties in metal matrix composites (MMCs). Microstructural analysis provides essential insights into the quality and uniformity of particle distribution. During composite fabrication, ensuring homogeneous dispersion in the melt and minimizing particle agglomeration or segregation during pouring and solidification are critical^1,3,5^. In the present study, the distribution of individual and hybrid reinforcements (SiC and B₄C) in the Al6061 matrix is examined using SEM and EDX techniques. Figures 3a–e present the SEM micrographs and corresponding EDX spectra of as-cast AL2B1S hybrid composites at various locations. Spectra obtained from identified regions (Spectrum 1, 2, and 3) confirm the presence of SiC, B₄C, and the Al6061 matrix. The micrographs show no signs of particle agglomeration, blow holes, or porosity, indicating a uniform distribution.

### Hardness measurement

The as-cast (AC) Al 6061 alloy exhibits a hardness of 80 VHN. Similarly, the AC monolithic and hybrid composites show enhanced hardness values, as presented in Table 3. In monolithic composites, increasing the reinforcement weight% leads to higher VHN values, primarily due to the uniform dispersion of reinforcement particles. The hardness values of heat-treated samples, including error margins from the average of three measurements, are also reported in Table 3. Among all compositions, hybrid composites display the highest hardness, with aging treatment further increasing the values due to the formation of metastable phases. Similar hardness enhancement trends have been reported for Aluminum hybrid composites reinforced with B₄C and carbon-based additives^1,4,5^.

Overall, the results confirm that both reinforcement addition and heat treatment have a significant influence on the hardness of Al 6061 composites. Among the various heat treatment conditions, the M1-treated samples exhibit the highest hardness compared to M2, S2, and S1. Our earlier work^19^ provides detailed microstructural characterization (SEM, EDS, XRD), confirming the presence and uniform distribution of SiC and B₄C reinforcements. The multistage solutionized (MSS) specimens consistently show higher hardness than single-stage solutionized (SSS) specimens. This improvement is primarily due to the complete homogenization and dissolution of secondary solute-rich phases during MSS treatment. In contrast, SSS specimens retain undissolved secondary phases, which contribute to slightly higher hardness immediately after quenching but limit long-term precipitation effectiveness. MSS specimens form fine, uniformly distributed, coherent precipitates upon aging, which significantly enhance hardness. This effect is less prominent in SSS specimens due to incomplete phase dissolution. According to Han et al.^17^, room-temperature secondary phases, such as Al₂Cu and Al₅Mg₈Cu₂Si₆, degrade mechanical performance. These phases do not dissolve fully under SSS at 495 °C for 8 h. However, MSS facilitates complete dissolution, forming a supersaturated solid solution that allows fine precipitation during aging. Additionally, MSS refines the morphology of eutectic silicon particles, transforming them from coarse and faceted to more spherical shapes, which further contributes to improved mechanical properties. These findings align with the results reported by Sharma et al.^21^.

**Table 3:** Hardness values of AC and heat-treated Al6061 and its composites (SSS + aging at 100 °C - S1, SSS + aging at 200 °C - S2, MSS + aging at 100 °C - M1, MSS + aging at 200 °C - M2]).

Sample	Heat treatment								
	AC	S1		S2		M1		M2	
	VHN (± 5)	VHN(± 5)	PAT in (h)	VHN(± 5)	PAT in (h)	VHN(± 5)	PAT in (h)	VHN(± 5)	PAT in (h)
Al 6061	80	105	13	99	11.5	140	16	126	14
AL1B	99	118	11	110	9.5	155	14	136	12
AL2B	101	127	10	117	8.5	170	12.5	150	11.5
AL3B	104	135	9	125	7.5	186	11	165	10.5
AL1S	96	109	10	104	8	145	12	130	9.5
AL2S	99	115	9	111	7.5	153	11.5	135	9
AL3S	100	122	8	113	6.5	167	10.5	145	8.5
AL1B2S	106	152	7.5	141	6.5	192	9	177	8.5
AL1.5B1.5 S	110	160	7	149	6	205	8.5	184	8
AL2B1S	118	172	6	162	5	222	8	206	7

### Ultimate tensile strength

**Fig. 4:**
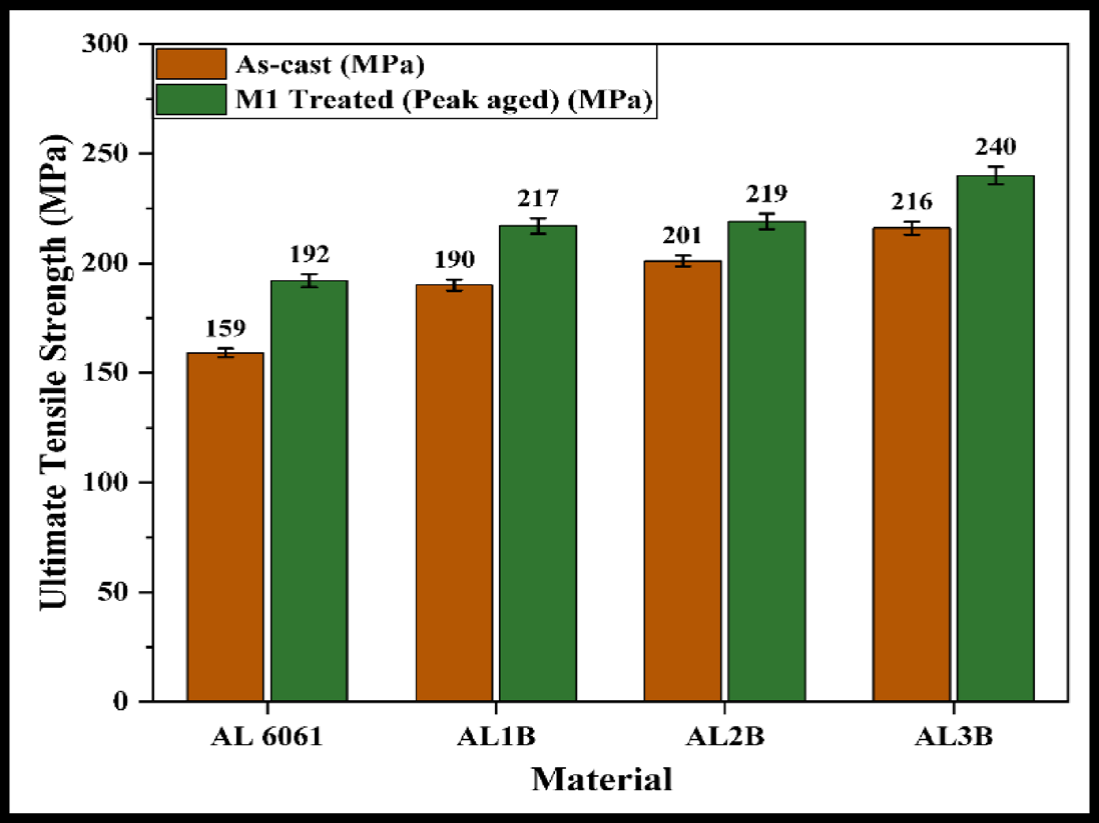
UTS of AC and M1 treated Al 6061 + B_4_C composite samples.

**Fig. 5:**
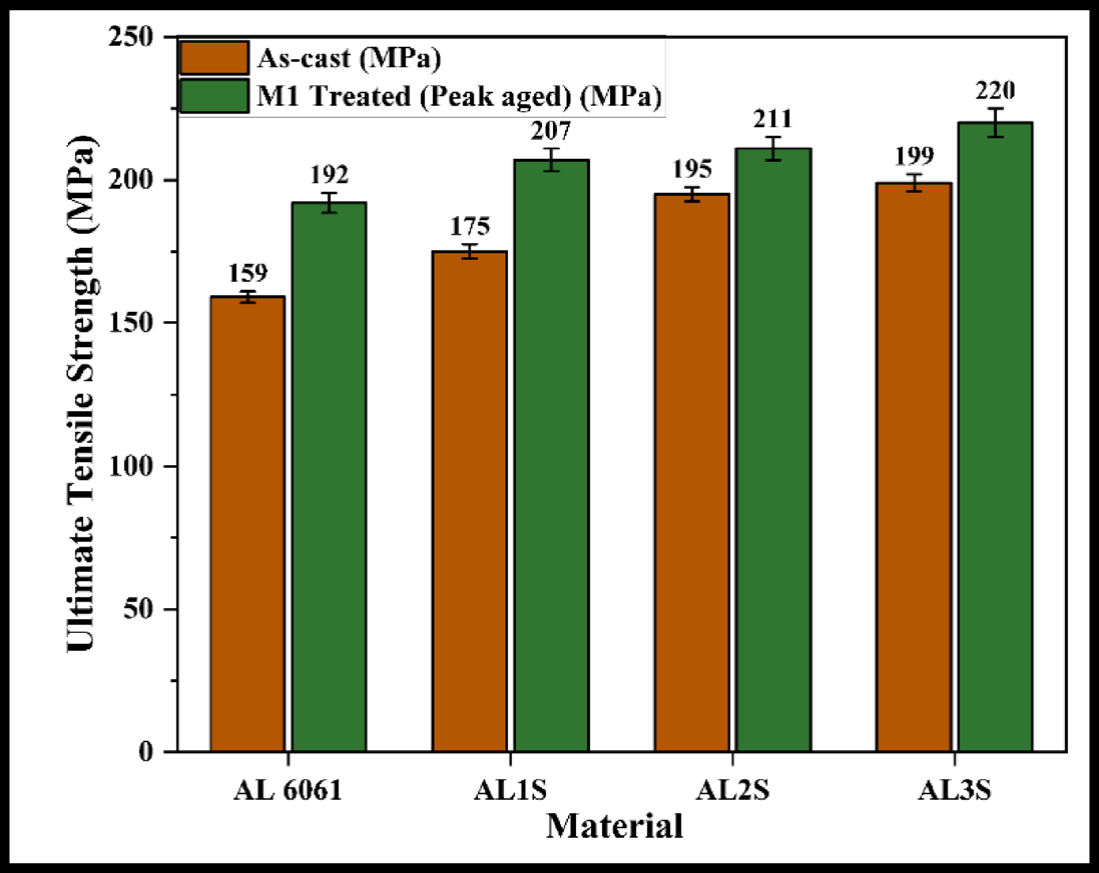
UTS of AC and M1 treated Al 6061 + SiC composite samples.

**Fig. 6:**
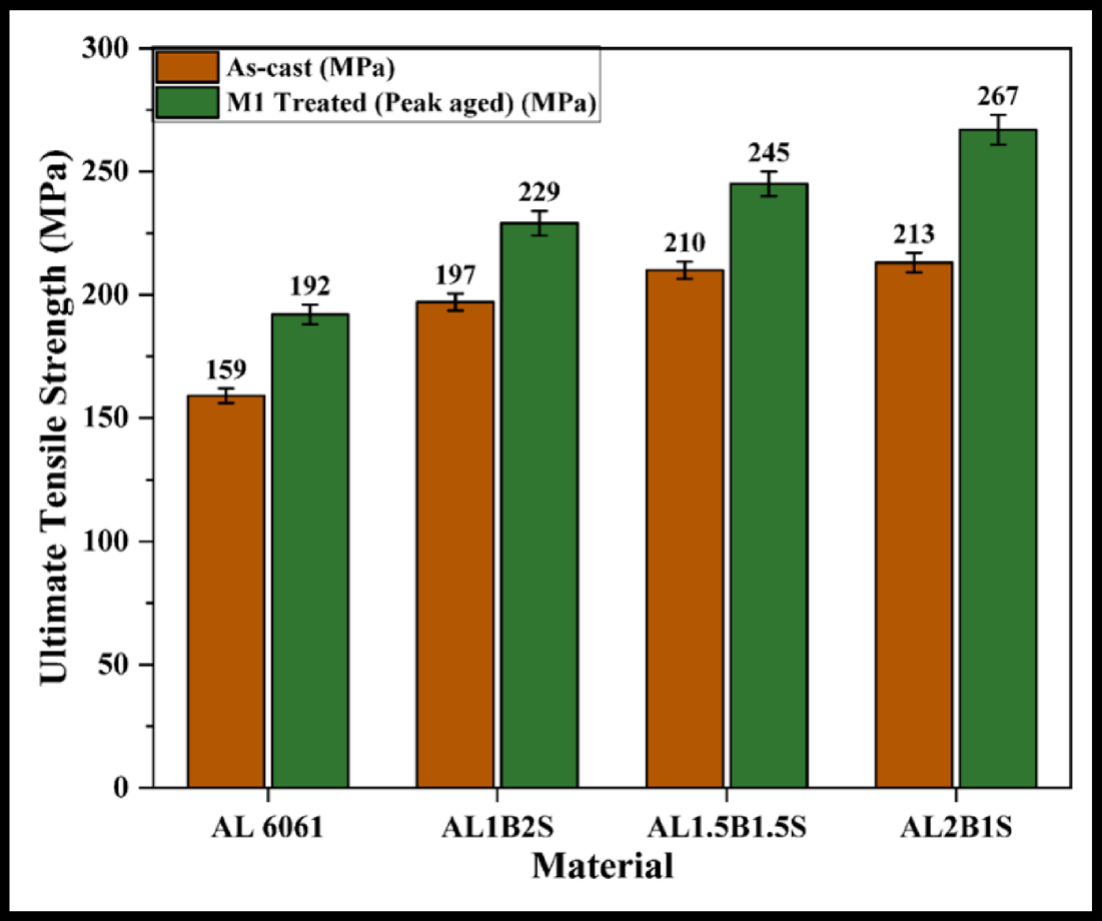
UTS of AC and M1 treated Al6061 + B_4_C + SiC composite samples.

The M1-treated samples exhibit the highest hardness values; therefore, only these samples are considered for further analysis and comparison. Tensile tests are conducted on the AC and M1 samples of the alloy and its composites. Figures 4, 5 and 6 present the UTS values for the Al 6061 + B₄C, Al 6061 + SiC, and Al 6061 + B₄C + SiC composite samples.

The observed increase in UTS for the composites, compared to the Al 6061 alloy, is primarily attributed to the presence of hard reinforcements, the effects of aging heat treatment, and the formation of intermetallic precipitates. These factors collectively contribute to an increase in dislocation density within the matrix. The elevated dislocation density enhances the nucleation and growth of secondary solute-rich precipitates during aging, effectively hindering dislocation movement and thereby strengthening the material.

Additionally, the hard reinforcement particles obstruct the plastic flow of the ductile matrix under tensile loading, leading to dislocation pile-up and localized stress concentrations. This, in turn, promotes work hardening and solid solution strengthening. These strengthening mechanisms, when combined or acting synergistically, result in a notable improvement in UTS, particularly in the hybrid composite^1–5,9,12,21–24^.

**Fig. 7:**
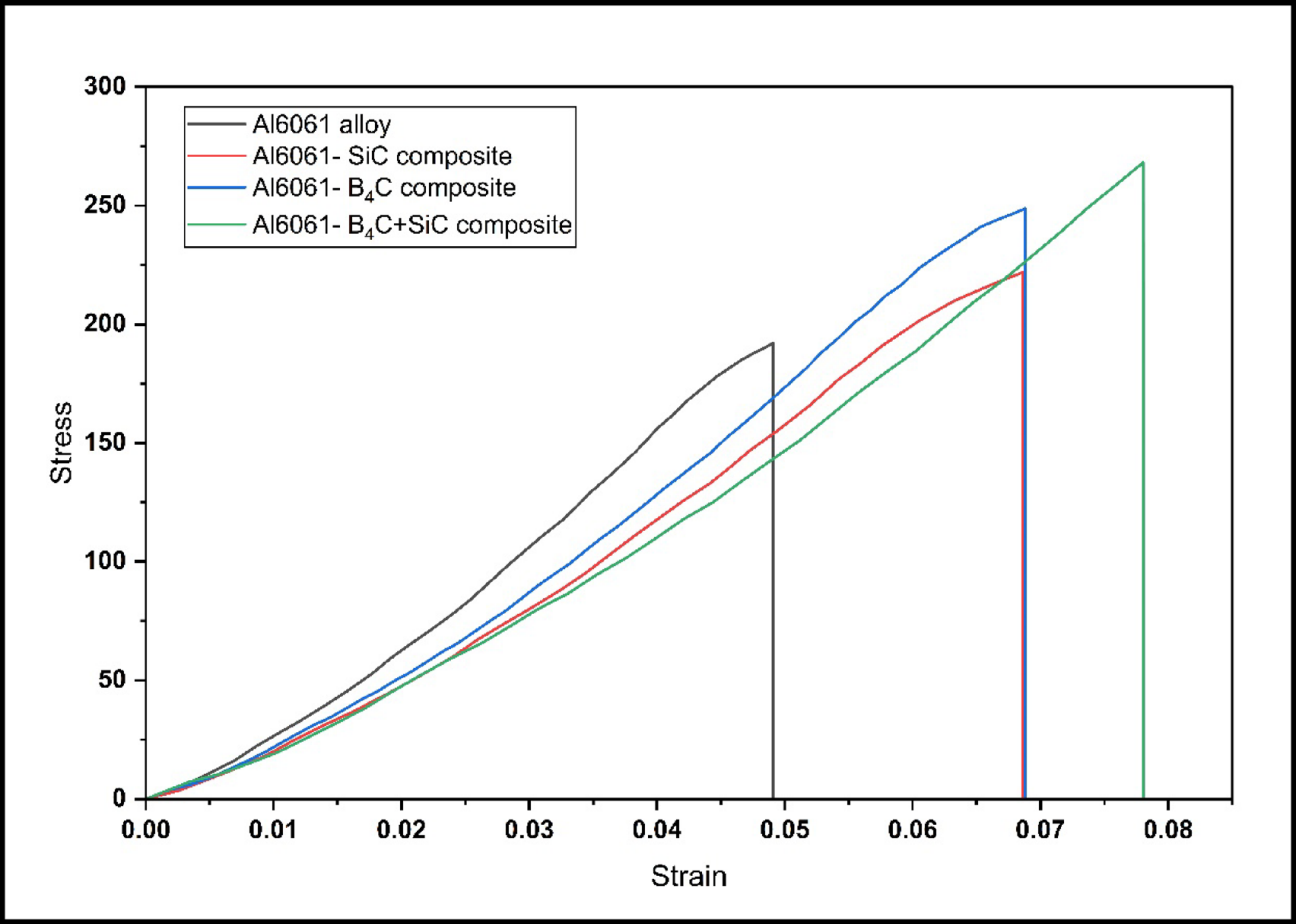
Stress-strain behavior of peak-aged Al6061 alloy and reinforced with SiC, B₄C, and SiC + B₄C composites.

Figure 7 presents the stress-strain behavior of peak-aged Al6061 and its composites reinforced with SiC, B₄C, and a hybrid combination of SiC + B₄C particles, highlighting significant variations in mechanical performance. The unreinforced, peak-aged Al6061 alloy exhibits a relatively ductile curve, characterized by moderate tensile strength and high elongation.

With the incorporation of SiC particles, the stress-strain curve shifts upward, indicating an increase in tensile strength due to improved load-bearing capability and effective stress transfer across the matrix-reinforcement interface. However, this improvement is accompanied by a noticeable reduction in ductility, resulting from particle-induced embrittlement and dislocation pile-up at the particle-matrix boundary^23,24^.

The Al6061-B₄C composite shows a similar trend, achieving even higher peak strength than the SiC-reinforced composite. This enhancement is attributed to the higher hardness and lower density of B₄C, along with its relatively better interfacial compatibility, which helps retain slightly more ductility.

Among all samples, the hybrid Al6061-SiC + B₄C composite exhibits the highest tensile strength, indicating a synergistic strengthening effect from the dual reinforcements. However, this is accompanied by a further reduction in ductility, as the combined presence of hard particles intensifies brittleness and limits plastic deformation. The stress-strain curve of the hybrid composite is steep and terminates abruptly, reflecting premature failure under tensile loading.

### Fracture surface analysis of al 6061 alloy and its composites

**Fig. 8:**
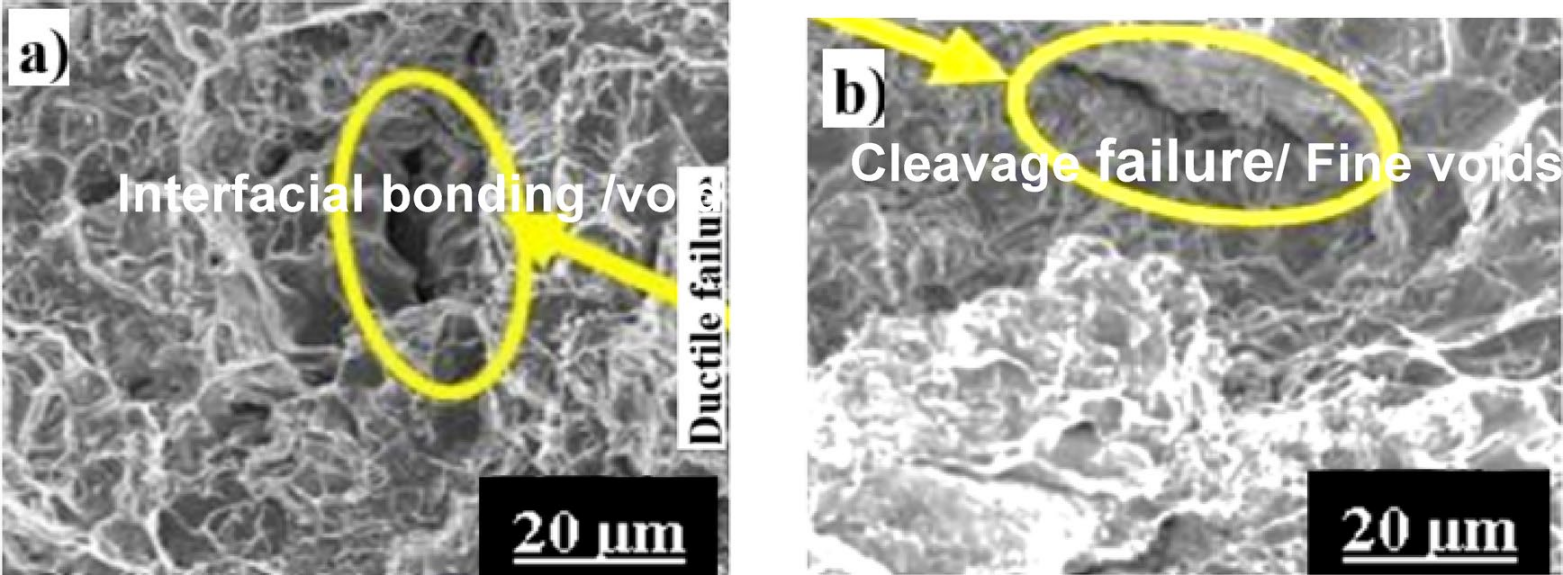
FSA of AC (**a**) and M1-treated (**b**) Al6061 samples.

Al6061 alloy samples SEM is employed to examine the fracture surfaces (FS) of tensile-tested samples. Figure 8 illustrates the FS of Al6061 in both AC and M1 conditions. The fracture surface of the AC-Al 6061 sample (Fig. 8a) reveals numerous fine, equiaxial dimples, indicative of a ductile fracture through dimple rupture. At higher magnification, characteristic cuplike depressions further confirm this failure mode. Micro-voids are visible near grain boundaries, and the presence of river patterns supports the predominance of ductile fracture. These microstructural features explain the relatively lower UTS values observed in AC-Al6061 samples.

In contrast, the fracture surface of the M1-treated Al6061 sample (Fig. 8b) displays a higher density of smaller, more uniformly distributed dimples, suggesting the presence of a greater number of finely spaced micro-voids. The reduced dimple size in peak-aged specimens correlates with enhanced tensile strength and ductility^25^, resulting in significantly higher UTS values compared to the untreated Al6061 alloy^18^.

**Fig. 9:**
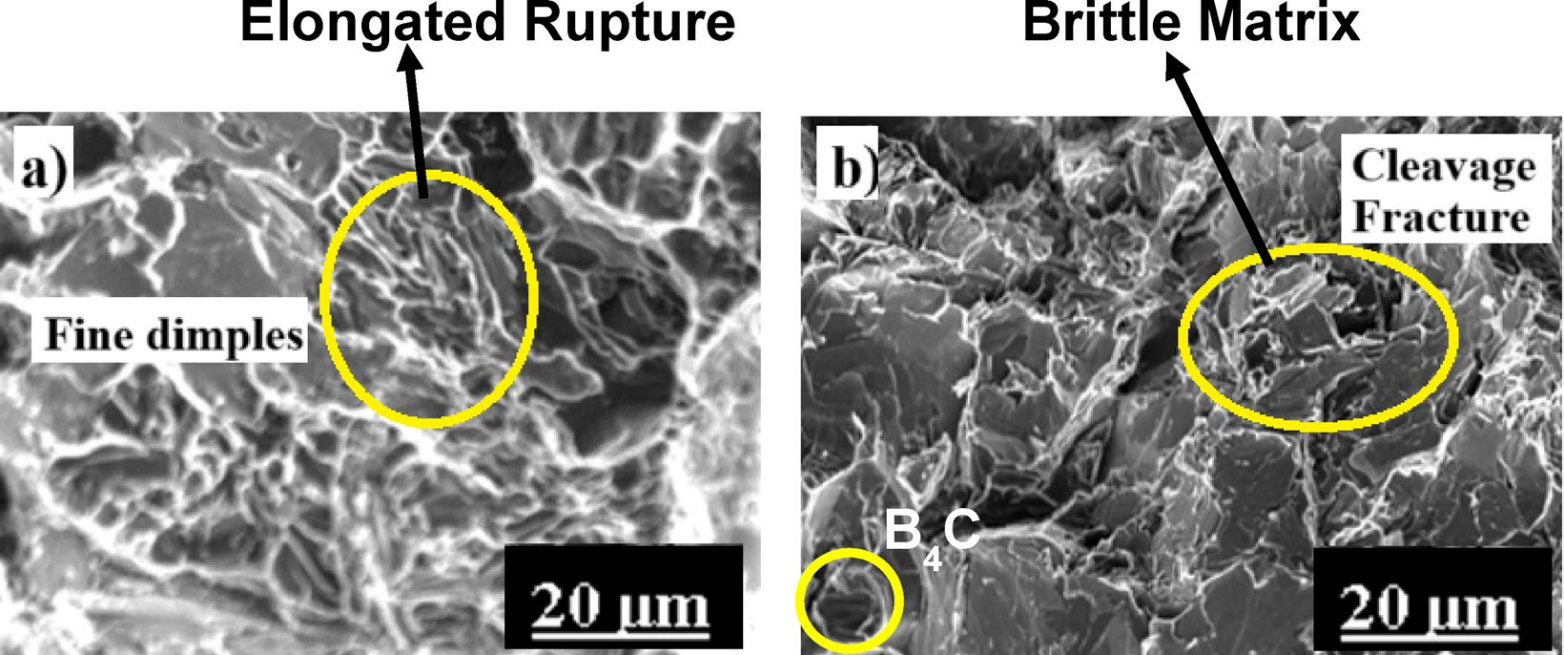
FSA of AC (**a**) and M1-treated (**b**) AL3B samples.

Figures 9a,b illustrates the fracture surfaces of AC- and M1-treated Al6061 + B₄C (AL3B) composites. The M1-treated sample shows finer and more uniformly distributed dimples, confirming ductile fracture through micro-void nucleation, growth, and coalescence around inclusions and second-phase particles. The presence of elongated dimples and quasi-cleavage regions indicates a mixed ductile–brittle mechanism, consistent with previous observations by Herbert et al.^26^. The reduced dimple density and non-uniform morphology reflect localized strain hardening and load transfer from the softer matrix to the hard B₄C particles. B₄C reinforcement, due to its high hardness and stiffness, restricts plastic deformation and enhances dislocation density, resulting in improved strength but reduced ductility. Similar fracture characteristics in B₄C-reinforced Al6061 composites have been reported by Auradi et al.^11^ and Baradeswaran et al.^10^, validating the present observations that the multistage solutionized (M1) condition enhances interfacial bonding and refines fracture morphology.

**Fig. 10:**
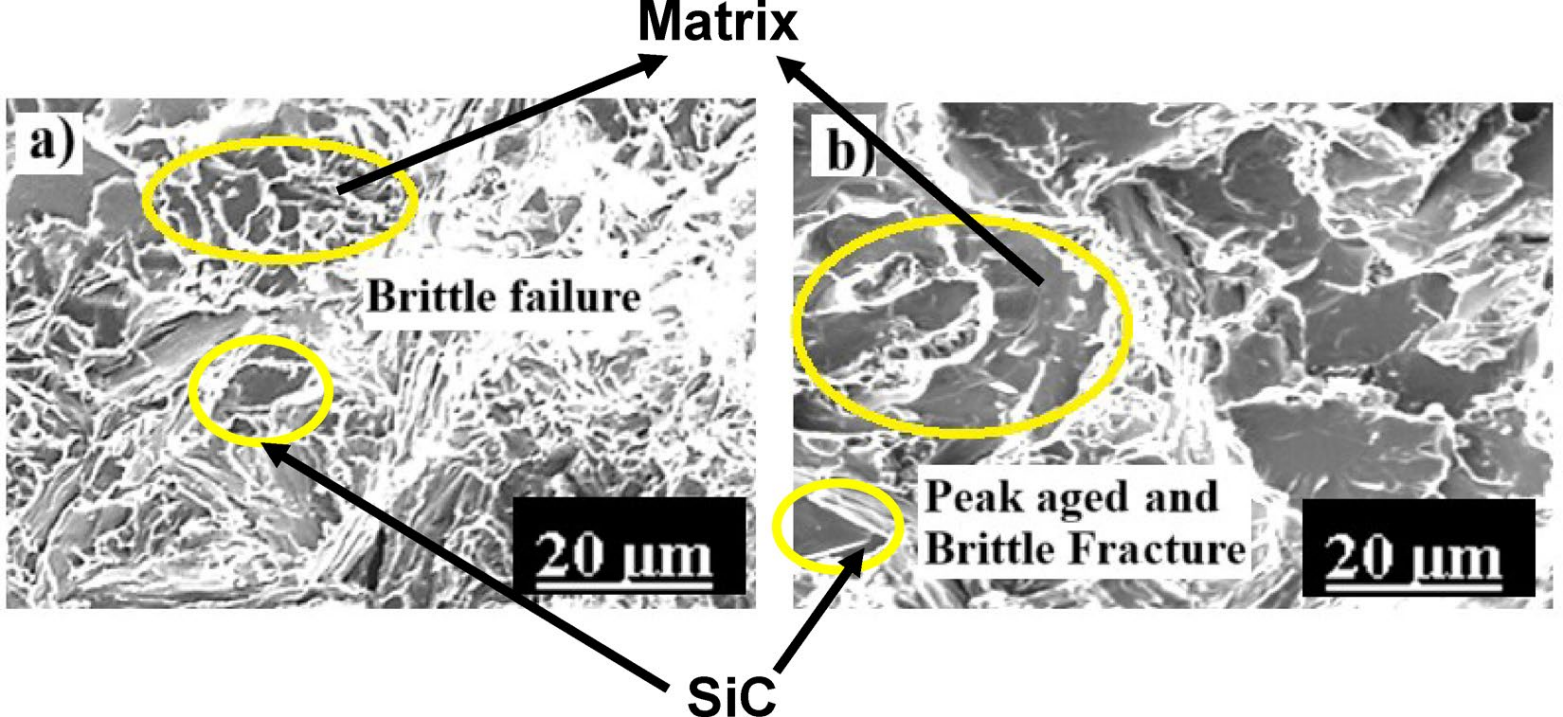
FSA of AC (**a**) and M1-treated (**b**) AL3S samples.

Figures 10a,b shows the fracture surfaces of Al6061 + SiC (AL3S) composites under AC and M1 conditions. Compared to unreinforced Al6061, both samples exhibit smaller, shallower dimples and flatter regions, confirming a transition toward a brittle fracture mode. The presence of fractured SiC particles within the matrix suggests strong interfacial bonding and efficient load transfer during tensile deformation^27,28^. The reduced dimple density results from SiC’s high hardness and modulus, which restrict plastic flow and act as stress concentrators that promote micro-void nucleation and early crack formation^9,23^. Similar features have been reported for SiC-reinforced Al-alloy systems, where fine, uniformly distributed particles improve strength but decrease ductility^10,14,17^. After multistage solutionizing and aging (M1), the matrix exhibits refined precipitates and metastable Mg₂Si phases^18^, further reducing ductile tearing. Consequently, the AL3S-M1 sample displays enhanced brittleness and quasi-cleavage facets, consistent with prior findings on aged Al–SiC composites^27,29^.

**Fig. 11:**
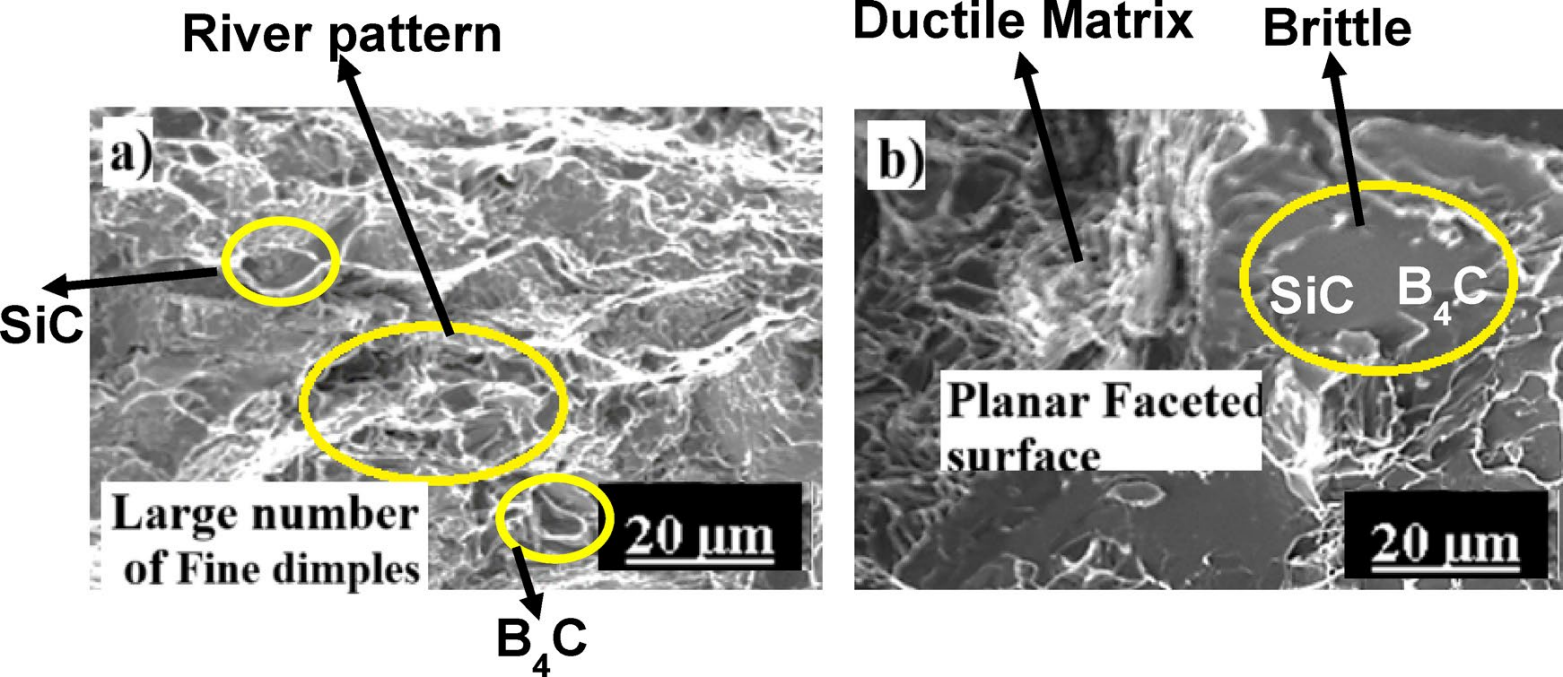
FSA of AC (**a**) and M1-treated (**b**) AL1B2S samples.

**Fig. 12:**
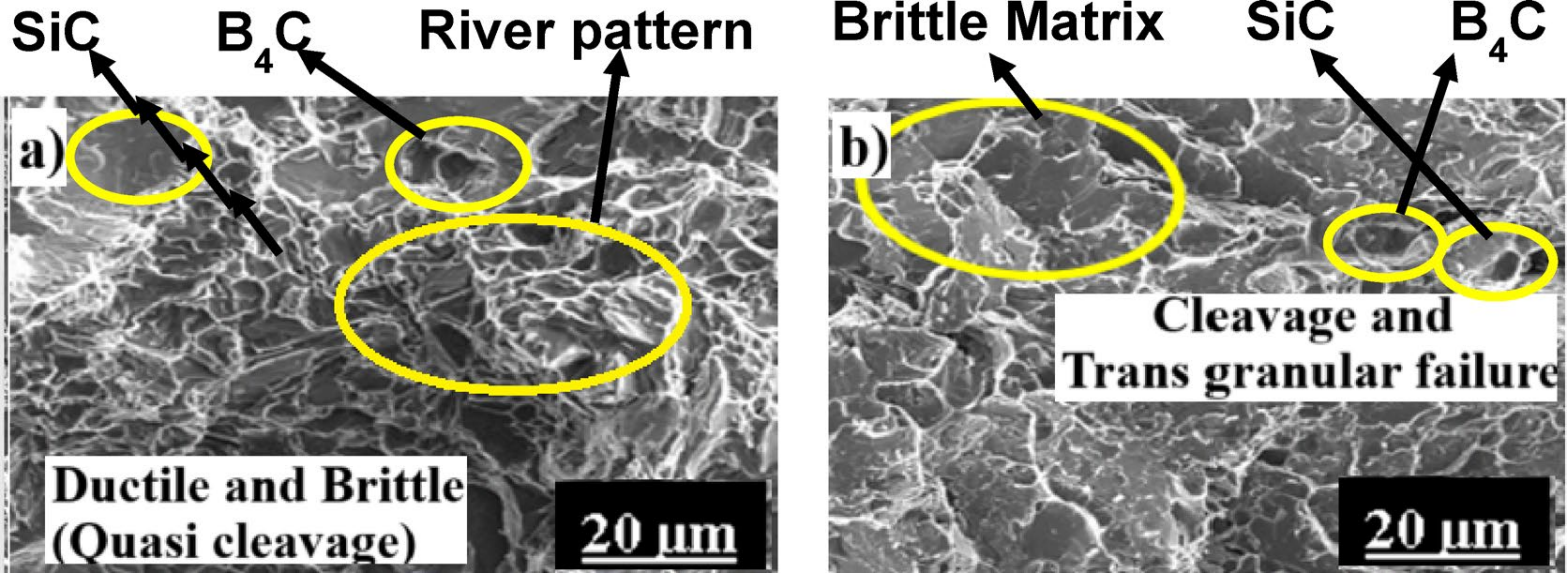
FSA of AC (**a**) and M1-treated (**b**) AL2B1S samples.

Al6061 + B_4_C + SiC samples The fracture surface analysis of AL1B2S-M1 and AL2B1S-M1 samples is shown in Figs. 11 and 12. Figure 11a presents the FS of the AC-treated AL1B2S composite, which exhibits reduced plasticity due to the incorporation of reinforcements. Following AAHT, plasticity decreases further, as shown in Fig. 11b. Debonding at the reinforcement–matrix interface leads to the formation of voids, cracks, and ultimately, failure.

The AC-AL1B2S sample demonstrates a predominantly ductile fracture mode, evidenced by the presence of dimples, necking, and tearing ridges. However, the appearance of planar facets also indicates regions of brittle failure. In contrast, the AL1B2S-M1 samples exhibit a mixed-mode fracture with a dominant brittle character. This transition is primarily attributed to grain refinement and the presence of hard intermetallic phases formed during the aging process.

Figures 12a,b depicts the fracture surfaces of AC- and M1-treated AL2B1S hybrid composites. The AC-treated sample shows mixed-mode fracture features with dimples, tear ridges, and localized brittle regions, whereas the M1-treated specimen exhibits pronounced quasi-cleavage and transgranular fracture facets. The increased brittleness in the hybrid composite is primarily due to the higher B₄C fraction, which elevates dislocation density and impedes plastic flow within the Al6061 matrix, consistent with the fracture morphology trends reported in recent Al hybrid composite systems^3,4^. Moreover, the thermal expansion mismatch between the ceramic reinforcements (B₄C and SiC) and the matrix generates residual stresses, which promote crack initiation along the particle–matrix interfaces^9,14^. Similar fracture morphologies have been reported in hybrid Al composites, where fine reinforcements enhance strength but reduce ductility due to particle-induced stress concentration and interfacial decohesion^10,22,30^. The quasi-cleavage features of the M1-treated sample confirm that multistage solutionizing and aging refine the matrix but increase brittleness through the formation of metastable precipitates^17,18,29^.

According to literature^30^, silicon and Mg₂Si phases are particularly prone to crack initiation and quasi-cleavage fracture. In the AL2B1S sample, fracture behavior is dominated by brittle mechanisms, with cracks frequently initiating at the matrix–reinforcement interface. Nevertheless, the fracture morphology also reveals a combination of ductile and brittle features, indicating a mixed-mode failure.

Closer examination shows regions with dimples, which reflect localized plastic deformation and are characteristic of the ductile nature of the Al 6061 matrix. In contrast, cleavage planes and particle pull-out sites highlight brittle fracture mechanisms, which are primarily influenced by hard reinforcement particles, such as SiC and B₄C. The interfacial bonding between the matrix and reinforcements is critical in governing fracture behavior. Strong interfacial bonding promotes ductile tearing and resists debonding, while weak interfaces lead to particle fracture and matrix separation.

A similar approach is demonstrated by Huang and Adityawardhana^31^, who correlated machine-learning-predicted fracture morphologies with experimental fractographic features of graphene-reinforced AZ91 composites. Their results validate that distinct morphological patterns, such as pull-out cavities and angular particle imprints, can reliably indicate the presence and fracture behavior of reinforcements even without localized EDS mapping. Overall, the observed mixed-mode fracture in AL2B1S arises from the complex interplay among microstructural features, reinforcement distribution, and the effects of heat treatment.

## Conclusions

- Precipitation hardening significantly enhances both the hardness and ultimate tensile strength of Al6061 alloy and its composites.
- Hybrid composites reinforced with B₄C and SiC display superior hardness compared to monolithic (single-reinforced) composites.
- The multistage solution heat treatment (MSS) combined with artificial aging (M1 condition) yields the best mechanical performance among all treatment conditions.
- Fracture surface analysis reveals that as-cast Al6061 primarily fails in a ductile mode, characterized by numerous fine dimples.
- Monolithic composites with B₄C or SiC alone exhibit mixed-mode failure, combining ductile and brittle characteristics.
- B₄C reinforcements contribute to both ductile and brittle features in the fracture surface.
- SiC reinforcements encourage a more brittle fracture mode in the composites.
- The synergistic combination of B₄C and SiC reinforcements, along with optimized heat treatment, effectively enhances both mechanical properties and fracture resistance, making the material suitable for high-strength applications.

## Data Availability

The corresponding author agrees to provide the data upon reasonable request.

## Abbreviations

Al–Mg-Si: Al 6061 alloy
UTS: Ultimate tensile strength
FSA: Fracture surface analysis
Al: Aluminum
PAT: Peak aged time-hr
SiC: Silicon carbide
B_4_C: Boron carbide
SSS: Single stage solutionizing
S1: SSS + AAHT 100˚C
M1: MSS + AAHT 100˚C
MSS: Multistage solutionizing
AAHT: Artificial aging heat treatment
VHN: Vickers hardness number
S2: SSS + AAHT 200˚C
M2: MSS + AAHT 200˚C
